# Evaluation of the Synuclein-γ (SNCG) Gene as a PPARγ Target in Murine Adipocytes, Dorsal Root Ganglia Somatosensory Neurons, and Human Adipose Tissue

**DOI:** 10.1371/journal.pone.0115830

**Published:** 2015-03-10

**Authors:** Tamara N. Dunn, Tasuku Akiyama, Hyun Woo Lee, Jae Bum Kim, Trina A. Knotts, Steven R. Smith, Dorothy D. Sears, Earl Carstens, Sean H. Adams

**Affiliations:** 1 Graduate Group in Nutritional Biology, University of California Davis, Davis, California, United States of America; 2 Department of Nutrition, University of California Davis, Davis, California, United States of America; 3 Department of Neurobiology, Physiology and Behavior, University of California Davis, Davis, California, United States of America; 4 National Creative Research Initiatives Center for Adipose Tissue Remodeling, Department of Biological Sciences, Institute of Molecular Biology and Genetics, Seoul National University, Seoul, Republic of Korea; 5 Arkansas Children’s Nutrition Center and Department of Pediatrics, University of Arkansas for Medical Sciences, Little Rock, Arkansas, United States of America; 6 Sanford-Burnham Medical Research Institute and Translational Research Institute, Florida Hospital, Orlando, Florida, United States of America; 7 Department of Medicine, University of California San Diego, San Diego, California, United States of America; University of Minnesota - Twin Cities, UNITED STATES

## Abstract

Recent evidence in adipocytes points to a role for synuclein-γ in metabolism and lipid droplet dynamics, but interestingly this factor is also robustly expressed in peripheral neurons. Specific regulation of the synuclein-γ gene (*Sncg*) by PPARγ requires further evaluation, especially in peripheral neurons, prompting us to test if *Sncg* is a *bona fide* PPARγ target in murine adipocytes and peripheral somatosensory neurons derived from the dorsal root ganglia (DRG). *Sncg* mRNA was decreased in 3T3-L1 adipocytes (~68%) by rosiglitazone, and this effect was diminished by the PPARγ antagonist T0070907. Chromatin immunoprecipitation experiments confirmed PPARγ protein binding at two promoter sequences of *Sncg* during 3T3-L1 adipogenesis. Rosiglitazone did not affect *Sncg* mRNA expression in murine cultured DRG neurons. In subcutaneous human WAT samples from two cohorts treated with pioglitazone (>11 wks), *SNCG* mRNA expression was reduced, albeit highly variable and most evident in type 2 diabetes. Leptin (*Lep*) expression, thought to be coordinately-regulated with *Sncg* based on correlations in human adipose tissue, was also reduced in 3T3-L1 adipocytes by rosiglitazone. However, *Lep* was unaffected by PPARγ antagonist, and the LXR agonist T0901317 significantly reduced *Lep* expression (~64%) while not impacting *Sncg*. The results support the concept that synuclein-γ shares some, but not all, gene regulators with leptin and is a PPARγ target in adipocytes but not DRG neurons. Regulation of synuclein-γ by cues such as PPARγ agonism in adipocytes is logical based on recent evidence for an important role for synuclein-γ in the maintenance and dynamics of adipocyte lipid droplets.

## Introduction

Metabolic homeostasis is maintained through a complex process involving cross-talk between different organ systems, and there is a growing body of evidence implicating associations or signals between white adipose tissue (WAT), brown adipose tissue (BAT) and the peripheral afferent nervous system [1–4]. Understanding the integration of these biological systems is important as it relates to energy balance and maintenance of a healthy body weight. Best understood is the involvement of the sympathetic nervous system (SNS) and its efferents in regulating adipose tissue lipolysis via catecholamine release [5]. For example, obesity is characterized by increased resting SNS activity and reduced SNS responsiveness to stimuli [6]. Additionally, peripheral nerves communicate afferent information to the central nervous system (CNS) related to the environment (i.e. temperature via somatosensory neurons) and nutritional status (i.e. nutrients and gut hormones via vagal afferents [7]), allowing for changes in energy storage and food intake to occur. Metabolic status, in particular obesity, insulin resistance and frank diabetes, can have a major impact on peripheral afferent nerve function. For instance, susceptibility to obesity on a high fat diet was associated with increased expression of orexigenic receptors that regulate food intake in vagal afferents in rats [8] and can lead to vagal leptin resistance [9]. Additionally, in rodent models of diet-induced obesity, somatosensensory neuropathies develop in the absence of overt hyperglycemia [10, 11], suggesting other metabolic factors besides glucose control can influence sensory nervous system biology. While there is a plethora of information regarding responses of WAT to metabolic cues and endocrine signals, less is known in this regard for molecular events in the peripheral nervous system (PNS). The driving molecular mechanisms that govern the relationship between metabolic status, adiposity, and PNS function are still being uncovered (recently reviewed in [12]), but transcription factors that are involved with metabolic responses may play a role. It is possible that some of these factors and their target genes are shared by adipose tissue and PNS neurons, which may coordinate integrated responses to changes in nutrition and metabolism.

PPARγ is a nuclear receptor critical for fat tissue development, adipocyte differentiation and lipid metabolism, and is modulated by certain fatty acid-derived ligands [13] and insulin-sensitizing thiazolidinediones (TZDs) such as rosiglitazone and pioglitazone. Interestingly, in recent clinical interventions involving type 2 diabetic patients reporting neuropathy, administration of TZDs resulted in improvements in gait and thermosensation [14, 15]. While these improvements may have been due to improved microvascular function via enhanced blood glucose control, it is plausible that activation of PPARγ via TZDs can directly influence somatosensory neuron proprioceptive and thermal sensory functions. Outside the context of glucose homeostasis, TZDs have been shown to be neuroprotective in response to various central nervous tissue insults (traumatic brain injury, Parkinson’s disease, Alzheimer’s, spinal cord injury), possibly by reducing microglial activation and NFκB activity, the latter resulting in a reduction of cytokine expression ([16]; see [17] for review). TZDs have also been demonstrated to attenuate neuropathic pain in response to peripheral nerve injury by similar mechanisms [18, 19]. Furthermore, neuronal PPARγ knockout mice (which lack the factor in neurons of both the PNS and CNS) have a distinct metabolic phenotype, being resistant to both diet-induced obesity and the insulin-sensitizing effects of TZDs [20], suggesting a possible role for PPARγ signaling in PNS or CNS neurons in metabolic homeostasis.

We have previously characterized two proteins that are uniquely co-expressed at high levels in adipocytes and peripheral afferent neurons: tumor suppressor candidate 5 (Tusc5) and synuclein-γ (*Sncg*; also known as BCSG1 or persyn) [21–23]. Synuclein-γ was traditionally characterized as a neuron marker found in sensory, sympathetic, and motor neurons as well as some CNS sites including the midbrain and cerebral cortex [24–26]. Synuclein-γ may play a role in tissue plasticity, since the synuclein-γ transcript and protein are induced in certain cancers, and synuclein-γ has been implicated in cancer progression, metastasis, and cell survival [27, 28]. Synuclein-γ over-expression in sensory neurons in culture disrupts neurofilament structure [24], a key component to neuronal plasticity, and high levels of synuclein-γ expression have been reported in the leading edges of re-growing rat axons [29]. The function of synuclein-γ in WAT is only recently coming to light. In human subcutaneous and visceral WAT, SNCG expression is increased in obese women [22], a phenotype that is typified by tissue remodeling and lipid storage. Recent studies in *Sncg* knockout mice have indicated an important role for this factor in maintenance of lipid droplet formation and regulation of lipolysis in adipocytes [30]. This lipid trafficking function may also be important in the nervous system: *Sncg* null mutant mice show alterations in their lipid composition and fatty acid patterns in brain tissue [31]. Furthermore, 48 hr of fasting downregulated synuclein-γ expression in the mouse paraventricular nucleus, a hypothalamic region involved in energy balance [32], suggesting that regulation of synuclein-γ expression or function in neurons could be sensitive to metabolic status.

Despite recent advances in understanding synuclein-γ function, little is known about the transcriptional regulation of this gene. There is some evidence to suggest that the *Sncg* gene locus is a PPARγ target, at least in adipocytes. A PPARγ response element (PPAR-RE, or DR-1 site) has been predicted (but not confirmed in functional tests) in the promoter region of the human *SNCG* gene [33], and we have previously demonstrated that short-term treatment with a non-TZD PPARγ agonist (GW1929) and a TZD (troglitazone) suppressed *Sncg* expression in differentiated murine 3T3-L1 adipocytes [22]. However, whether WAT synuclein-γ is indeed regulated by PPARγ *in vivo* in humans, and confirmation that PPARγ binds to the gene promoter have not been explored. Furthermore, nothing is known about the response of synuclein-γ expression to PPARγ agonism in the PNS. To this end, studies were conducted to more fully characterize the relationship between synuclein-γ and PPARγ activation in adipocytes, and to determine if, as seen previously with short-term treatment in murine adipocyte cell culture, PPARγ agonist treatment would decrease synuclein-γ mRNA expression in human adipose tissue in subjects treated with a TZD. We tested if synuclein-γ expression is coordinately regulated with the murine (*Lep*) and human (*LEP*) leptin genes, considering our prior observations that WAT synuclein-γ transcript expression changes were correlated with leptin expression in one human lean and obese cohort [22]. Results in adipose preparations were complemented by studies testing the novel hypothesis that as for adipocytes, TZD treatment would elicit a down-regulation of synuclein-γ expression in PNS neuron preparations derived from murine dorsal root ganglia (DRG).

## Materials and Methods

### 3T3-L1 adipocyte differentiation and PPAR γ agonist dose-response studies

The impact of exposure to PPARγ agonism on *Sncg* mRNA abundance was tested in the murine 3T3-L1 adipocyte model. Conditions were as described previously [23], except cells were grown in 6-well uncoated plates and the maintenance and treatment media contained 1 nM insulin. To establish the relationship between PPARγ agonism and synuclein-γ expression, mature adipocytes (10–12 days post-differentiation, media changed every 3–4 days) were cultured for 24 hr in media containing one of the following treatments with the doses indicated: (1) vehicle (dimethyl sulfoxide; 0.1% by volume), (2) rosiglitazone (Cayman Chemical), a TZD PPARγ agonist (10 nM, 100 nM or 1000 nM), or (3) the liver X receptor (LXR) agonist T0901317 (Cayman Chemical) (10 nM, 100 nM, or 1000 nM), a range of concentrations and time previously reported to increase lipid accumulation and basal glucose uptake in adipocytes as well as the expression of LXR target genes [34, 35]. Additional preparations included pretreatment with a PPARγ antagonist, T0070907 (Cayman Chemical) [36], for 30 minutes (10 μM) before co-exposure with rosiglitazone. This regimen was shown to be effective in reducing PPARγ agonist effects on known targets in pilot studies (data not shown). The experiment was replicated twice, with n = 4–8/experiment. RNA was prepared using Trizol-based methods for cell culture samples as per manufacturer’s instructions (Ambion, Austin, TX), and transcript abundances of target genes were measured as described under “Gene Expression Analyses” section below.

### Chromatin immunoprecipitation (chip) assays

ChIP assays were performed as described previously [37, 38]. In brief, confluent preadipocytes (day 0) and differentiated 3T3-L1 adipocytes (at day 4 or 8 following addition of differentiation cocktail, which was added over days 1–2) were cross-linked in 1% formaldehyde at 37°C for 10 minutes and resuspended in 200 μL of Nonidet P-40-containing buffer (5 mM PIPES, pH 8.0, 85 mM KCl, and 0.5% NP-40). Crude nuclei were isolated and lysed in 200 μL lysis buffer (1% SDS, 10 mM EDTA and 50 mM Tris-HCl, pH 8.1), and lysates were sonicated and diluted 10-fold with immunoprecipitation buffer (16.7 mM Tris-HCl [pH 8.1], 167 mM NaCl, 1.2 mM EDTA, 0.01% SDS, and 1.1% Triton X-100). Lysates were immunoprecipitated with anti-acetylated-histone H3 (K9) (1 μg), anti-acetylated-histone H4 (pan) (1 μg), or anti-PPARγ (1 g) antibodies for 12 hours at 4°C. Immune complexes were incubated with Protein A-Sepharose CL-4B (Amersham-Biosciences) for 2 hours at 4°C. “Input” represents 10% of the total input chromatin. After successive washings, immune complexes containing DNA were eluted and the precipitated DNA was amplified by PCR. This experiment was replicated three times. Promoter primer pairs used in this study are as follows: (1) 313 bp amplicon for putative −9.9 kb DR-1 site (5′-ATTGCCCAGGCGCTTGCAG, 3′:CAGGCTTTTACCCAGAGCG); (2) 140 bp amplicon for putative −3.4 kb DR-1 (5′:GCCTGCTACTGGGAGTAACG, 3′:TGATTCCATTCAGAGCCTCA); (3) nonspecific 210 bp amplicon near the putative −6.2 kb DR-1 sequence (5′-GACATTCCAGAGGACCCAGA, 3′-GGTGTCTGACTGCGTGTTTC). Antibodies: Ac-H3 K9 (Upstate 07–352), Ac-H4 pan (Upstate 06–866), PPARg (Abcam ab41928).

### SNCG WAT transcript abundance following treatment with TZDs in human subjects with type 2 diabetes mellitus (T2DM) and in non-diabetics

#### Cohort 1

Expression of WAT *SNCG* was examined in microarray results from a cohort of non-diabetic (n = 17) and type 2 diabetic (n = 8) pioglitazone (PIO)-treated subjects for which details on the experimental conditions have previously been published [39]. Clinical characteristics of these PIO-treated subjects are included in Table 1. Needle biopsies of abdominal subcutaneous adipose tissue were taken after a 10 hr overnight fast, flash-frozen in liquid nitrogen and stored at -80°C. After completing the baseline studies, subjects were treated for 12 wk with PIO (45 mg/d). Ethics Statement—The experimental protocol and consent materials were approved by the Institutional Review Board for Human Subjects of the University of California, San Diego, and subjects provided written informed consent.

**Table 1:** Clinical characteristics of adults before and after treatment with 45 mg/day pioglitazone (PIO) for 12 weeks (Cohort 1).

	Non-Diabetic		Type 2 Diabetic		
	n = 17		n = 8		
Ethnicity (C/NC)	15/2		6/2		
Gender (M/F)	14/3		8/0		Group Difference
	AVG	SEM	AVG	SEM	t-test
Age	48	3	48	3	ns
BMI_1_, kg/m^2^	30.1	1.9	36.5	2.8	ns
BMI_2_, kg/m^2^	30.5	2.0	37.9	2.7	#
BMI, change	ns		*		
Weight_1_, kg	89.3	5.5	111.3	8.5	#
Weight_2_, kg	90.5	5.5	115.7	8.6	#
Weight, change	ns		*		
FPG_1_, mg/dL	95	2	173	19	#
FPG_2_, mg/dL	90	2	132	13	#
FPG, change	*		*		
serum insulin_1_, μU/mL	16.5	2.1	19.7	3.6	ns
serum insulin_2_, μU/mL	12.9	1.6	11.8	1.0	ns
Insulin, change	*		*		
% HbA1c_1_	5.4	0.1	6.7	0.3	#
% HbA1c_2_	5.6	0.1	7.0	0.5	#
HbA1c, change	ns		ns		
R_d1_, mg/kg/min	7.8	0.9	3.4	0.6	#
R_d2_, mg/kg/min	8.1	0.6	5.5	0.6	#
R_d_, % change	13.4	7.7	83.1	33.0	#
R_d_, change	ns		*		
Tot Chol_1_, mg/dL	192	10	192	22	ns
Tot Chol_2_, mg/dL	183	13	175	14	ns
Tot Chol, change	ns		ns		
LDL_1_, mg/dL	125	8	123	22	ns
LDL_2_, mg/dL	131	9	110	15	ns
LDL, change	ns		ns		
HDL_1_, mg/dL	38	3	35	2	ns
HDL_2_, mg/dL	37	3	38	4	ns
HDL, change	ns		ns		
TG_1_, mg/dL	151	36	170	26	ns
TG_2_, mg/dL	99	11	155	22	#
TG, change	ns		ns		

Subscripts refer to pre-intervention_1_ or post-intervention_2_ with pioglitazone (PIO)

* p<0.05 in paired t-test pre- vs. post-treatment; ns, not significant

# p<0.05 in unpaired t-test non-diabetic vs. type 2 diabetic

C/NC—Caucasian, non-Caucasian; BMI—body mass index; FPG—fasting plasma glucose; HbA1c—hemoglobin A1c

R_d_—rate of glucose disposal during hyperinsulinemic euglycemic clamp (mg/kg per min)

Tot Chol—serum total cholesterol; TG—serum triglycerides

#### Cohort 2

Archived samples were available from a subset of a second cohort of volunteers who participated in a study previously described by Smith et al. [21, 40]. Subjects diagnosed with type 2 diabetes mellitus (T2DM) were treated with PIO (30 mg/day, n = 14: 5 male, 9 female) for 11–17 weeks. If fasting plasma glucose was >100 mg/dL or HbA1c was ≥7.0% at week 8 of the study, the dose of PIO was increased to 45 mg/d. Clinical characteristics of this cohort are provided in Table 2. Subcutaneous abdominal WAT biopsies were obtained by Bergstrom needle at the beginning and end of the study after an overnight fast, with local Lidocaine anesthesia, and samples were flash-frozen and stored at −80°C until processed for mRNA and target gene transcript quantitation as described below. Ethics Statement—Clinical study protocol and consent materials were approved by the Institutional Review Board of the Pennington Biomedical Research Center, and subjects provided written informed consent.

**Table 2:** Clinical characteristics of adults with T2DM before and after treatment with 30 mg/day pioglitazone (PIO) for >11 weeks (Cohort 2).

	n = 15	
Ethnicity (C/NC)	10/5	
Gender (M/F)	5/10	
	AVG	SEM
Age	58	1.9
BMI_1_, kg/m^2^	32.3	1.4
BMI_2_, kg/m^2^	33.7	1.5
BMI, change	*	
Weight_1_, kg	92.4	4.3
Weight_2_, kg	96.3	4.6
Weight, change	*	
FPG_1_, mg/dL	153.2	10.4
FPG_2_, mg/dL	129.8	6.6
FPG, change	*	
serum insulin_1_, μU/mL	13.7	2.5
serum insulin_2_, μU/mL	10.4	1.4
Insulin, change	ns	
HDL_1_, mg/dL	50.4	4.0
HDL_2_, mg/dL	58.3	4.2
HDL, change	*	
TG_1_, mg/dL	183.2	33.4
TG_2_, mg/dL	159.7	43.6
TG, change	ns	
Total Body Fat_1_ (kg)	33.7	2.6
Total Body Fat_2_ (kg)	37.2	2.9
Fat Mass, Change	*	
Total Body Lean Mass_1_ (kg)	58.4	3.6
Total Body Lean Mass_2_ (kg)	58.7	3.4
Lean Mass, Change	ns	
Fat Mass_1_ (%)	36.7	2.2
Fat Mass_2_ (%)	38.7	2.2
Fat Mass (%), Change	*	

Subscripts refer to pre-intervention_1_ or post-intervention_2_ with pioglitazone (PIO)

* p<0.05 in paired t-test pre- vs. post-treatment; ns, not significant

C/NC—Caucasian, non-Caucasian; FPG—fasting plasma glucose; TG—serum triglycerides

Total body fat and lean masses measured by DEXA

### DRG primary culture studies

The changes in *Sncg* mRNA expression in peripheral sensory neurons in response to PPARγ agonism was tested using primary mouse dorsal root ganglia (DRG). Cells were isolated and grown as previously described [41], derived from *ad lib* fed 7–10 wk old C57BL/6 male mice fed laboratory chow (Harlan, #2918) and housed under standard conditions (22°C, 12:12 light:dark cycle). Four hours after plating, cells were incubated with either vehicle (dimethyl sulfoxide; 0.1% by volume) or 100 nM rosiglitazone for 24 hours. This concentration represents a high effective level for regulation of adipocyte target genes (see Results), and also within the range previously reported to induce gene transcription in primary rat DRG [42]. The experiment was replicated 4 times, with n = 4–6/experiment. RNA was prepared using Trizol-based methods for cell culture samples as per manufacturer’s instructions (Ambion, Austin, TX) using Microscale RNA filter cartridges (Cat# Am10066G), and transcript abundances of target genes were measured as described under “Gene Expression Analyses” section below. Animal studies were approved by the University of California, Davis, Institutional Animal Care and Use Committee.

### Gene expression analyses

RNA was prepared from 3T3-L1 adipocytes and human WAT biopsy samples using a Ribopure kit (Applied Biosystems-AM1924). Primary cultured mouse DRG RNA was extracted using micro-Ribopure filters (AB AM1931). In human WAT biopsy samples from Cohort 2, surface oil was removed from retroperitoneal (RP)-WAT homogenates prior to extraction to discourage lipid contamination and improve the quality of the RNA. RNA abundance was quantified using a NanoDrop ND-1000 Spectrophotometer (NanoDrop Technologies), and cDNA was synthesized from total RNA using Superscript III reverse transcriptase (Invitrogen) followed by RNase-H treatment as per the manufacturer’s instructions. Gene expression analyses by quantitative PCR utilized gene-specific Taqman primers and FAM-MGB labeled probes (Assays-on-Demand, Applied Biosystems, Inc.) and were analyzed in duplicate or triplicate for each sample using an ABI 7900HT instrument. Reactions were carried out in a 384-well format containing the following in each well: cDNA corresponding to 7 ng of original total RNA (3T3-L1 PPAR dose response studies), 5 ng (human WAT samples); cDNA was dried in each well prior to adding qPCR reagents to facilitate an 8 μL/well assay. Wells also contained 1x Master Mix (ABI Universal PCR Master Mix, part no. 4309169) and 1x specific primer-probe mix. Cycle conditions were 50°C for 2 minutes, 95°C for 10 minutes, 40 cycles of 95°C for 15 s/60°C for 1 minute. Amplification cycle number (Ct) of control mRNA (eukaryotic 18S) for cell culture experiments, or pre treatment expression (PPIB) for human adipose was determined using commercial primers and probes (ABI) to correct for template loading differences, and expression values were determined relative to treatment control transcript levels using a mathematical formula as previously described [23]. Primers/probes ABI identifiers for mouse studies were *Sncg* (Mm00488345_m1), *Fabp4* (Mm00445880_m1), *Pparg* (Mm00440945_m1), *Lep* (Mm00434759_m1), *Calca* (Mm00801463_g1). For human tissue studies, identifiers were SNCG (Hs00268306_m1), LEP (Hs00174877_m1), PPIB (Hs00168719_m1).

### Statistics

Dose-response data for cell culture experiments were evaluated by 1-way ANOVA with a *post hoc* Dunnett’s test comparing groups to the vehicle-treated control (PrismGraph 6.0, GraphPad, San Diego, CA). ROUT Test (Graphpad Prism V.6) was utilized to identify and exclude outliers. Effect of PPARγ antagonist was evaluated by Student’s *t*-test for within each concentration of rosiglitazone. To assess differences in gene transcript levels following rosiglitazone treatment in primary cultured DRG cells, unpaired *t*-tests were used. To assess differences in gene transcript levels of human WAT for pre- and post- PIO-treated groups, paired *t*-tests were used. The relationship between synuclein-γ and leptin mRNA abundances was tested by Pearson correlation statistic (Graphpad Prism V.6). Means ±SEM are presented, and P<0.05 was considered statistically significant.

## Results

### Effects of PPARγ agonism (rosiglitazone) on expression profiles in mature 3T3-L1 adipocytes

**Fig 1:**
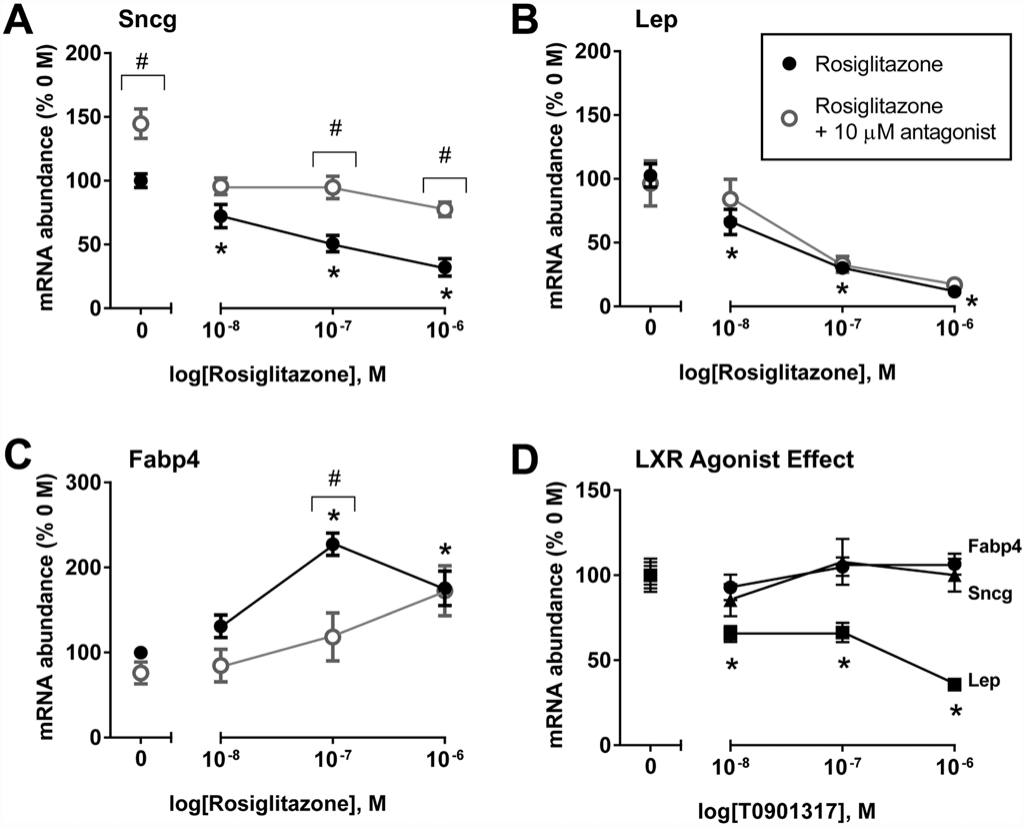
Regulation of mRNA expression levels for synuclein-γ (Sncg) (A), leptin (Lep) (B), and fatty acid binding protein (Fabp4) (C) by 24 hr short term treatment with the PPARγ agonist rosiglitazone, or co-treatment with PPARγ antagonist, T0070907, in mature 3T3-L1 adipocytes. Also shown are mRNA expression levels following treatment with LXR agonist, T0901317, in mature 3T3-L1 adipocytes **(D)**. Values are means +/- SEM, n = 7–15 per concentration; data from two experiments. Transcript level in vehicle-treated (0 M) control cells was considered 100%; gene expression values were calculated within experiment relative to control. *P<0.05, 1-way ANOVA with a post hoc Dunnett’s test comparing rosiglitazone concentrations to the vehicle-treated control. #P<0.05, Student’s t test for effect of 10 μM T0070907 antagonist at each individual concentration of rosiglitazone. Raw data is provided in S1 Data.

Previously we reported that 18 hr treatment of murine 3T3-L1 adipocytes with the non-TZD PPARγ agonist (GW1929) or with troglitazone decreased *Sncg* expression in fully-differentiated adipocytes, and this effect followed the same trend for the *Lep* (leptin) gene transcript [22]. To evaluate whether *Sncg* regulation is truly via PPARγ activation and not off-target effects, a dose-response study was conducted in mature 3T3-L1 adipocytes using a different TZD drug (rosiglitazone), with or without a PPARγ antagonist T0070907. The expression of known PPARγ target genes, such as *Fabp4* (fatty acid binding protein 4) and *Lep* (leptin) were tested as positive controls for PPARγ activation, since they are up- and down-regulated by such treatment, respectively [43–45]. As expected, *Fabp4* transcript levels showed significant dose-dependent increases to 24 hr treatment with rosiglitazone (at 100 nM and above), while leptin transcript showed a significant decrease at 100 nM and 1000 nM (Fig. 1B and 1C). As with leptin, rosiglitazone significantly decreased *Sncg* mRNA abundance (Fig. 1A). To further explore the specificity of the TZD effect, 3T3-L1 adipocyte cells were co-treated with the PPARγ antagonist T0070907 and rosiglitazone. Co-treatment with PPARγ antagonist significantly blunted roziglitazone-induced reductions in *Sncg* gene expression and induction of *Fabp4* (Fig. 1A, C), but had minimal effect on *Lep* expression (Fig. 1B). Interestingly, PPARγ antagonism alone (T0070907 + Vehicle) significantly increased *Sncg* mRNA expression levels by 50%, suggesting that basal PPARγ activity, either due to constitutive ligand-independent activity of the AF-1 site or the high levels of endogenous ligands in adipocytes [46], may act as a governor on *Sncg* expression.

It has been reported that PPARγ activation can have both LXR-dependent and LXR-independent actions; LXR activation can be a downstream event following PPARγ activation [47, 48]. Additional specificity studies were conducted using the LXR agonist, T0901317. LXR agonism had no effect on the gene expression of *Sncg* or *Fabp4* (Fig. 1D), excluding LXR as an important mediator of these genes in adipocytes. In contrast, *Lep* expression was significantly reduced by LXR agonist treatment at all concentrations (Fig. 1D). This highlights that multiple factors can regulate the *Lep* gene, and suggests that the effect on *Lep* expression following PPARγ activation is, at least in part, LXR-dependent.

### ChIP results

**Fig 2:**
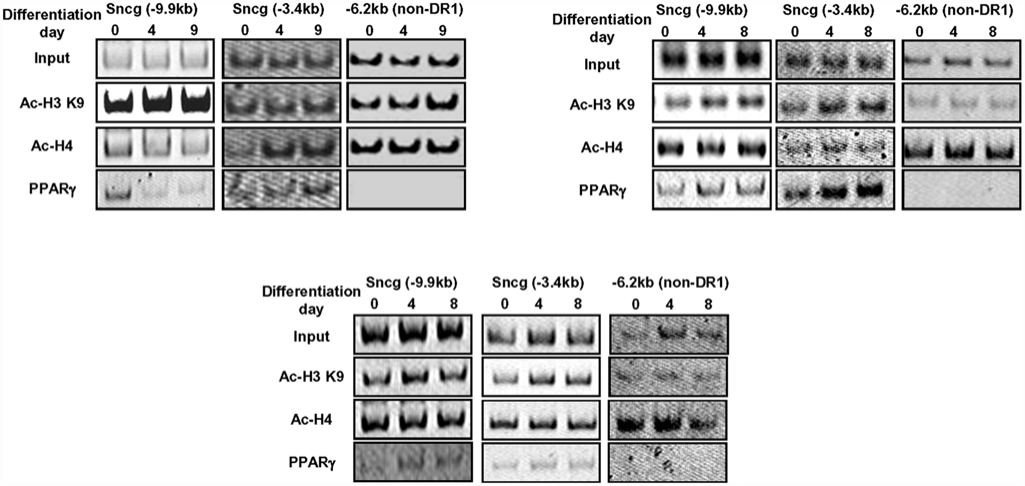
Temporal changes in PPARγ binding at predicted DR1 sites in the promoter region of the murine *Sncg* gene during 3T3-L1 adipocyte differentiation. ChIP studies were performed in pre-adipocytes and maturing adipocytes at days 4 and 8 post-differentiation initiation, employing anti-PPARγ antibody and sequence-specific primers for putative DR1 sites (PPAR-response elements) located at promoter regions—9.9 and—3.4kb, and a non-DR1 site at-6.2 kb, relative to the murine *Sncg* start codon. Also shown are histone acetylation patterns at each site, employing anti-acetylated histone-3 K9 (Ac-H3 K9) and anti-acetylated histone-4 pan (Ac-H4). Images are results from 3 independent experiments. Amplicon sizes are described in the text, and a representative image showing DNA band sizes is provided as S1 Fig.

To confirm that PPARγ protein binds to the murine *Sncg* gene promoter region during adipocyte differentiation in 3T3-L1 cells, ChIP analyses were focused at two potential binding sites with DR1 sites, before differentiation (day 0) and at 4 and 8 d post-initiation of adipocyte differentiation. PPARγ protein binding was observed at two predicted DR1 sites at-9.9 kb and-3.4 kb at all time points, but no binding was detected at a non-DRI promoter site (Fig. 2). Although histone acetylation is thought to be an important factor in promoting accessibility of DNA to transcription factors at the level of chromosome, it appears that there were no distinct differences in the magnitude of histone acetylation at the PPARγ-binding sites versus the non-DR-1 region in the *Sncg* gene (Fig. 2). *Sncg* expression is typically induced during 3T3-L1 adipocyte maturation and especially apparent several days post-differentiation [22]. Since we observed PPARγ binding to the *Sncg* promoter at all stage of adipogenesis, there must be a complex regulation of *Sncg* by PPARγ for which promoter binding alone may not fully explain adipogenesis-induced *Sncg* expression.

### SNCG expression in WAT of humans treated with TZDs

Previously, we observed that synuclein-γ and leptin transcript expression levels appeared to be co-regulated in human WAT [22]. To confirm this finding in new human cohorts and to determine if synuclein-γ and leptin are regulated by PPARγ agonists *in vivo*, we leveraged archived tissue samples from two separate clinical studies to investigate whether treatment with the TZD pioglitazone (PIO) would alter mRNA expression of these genes in human subcutaneous WAT.

#### Cohort 1

**Fig 3:**
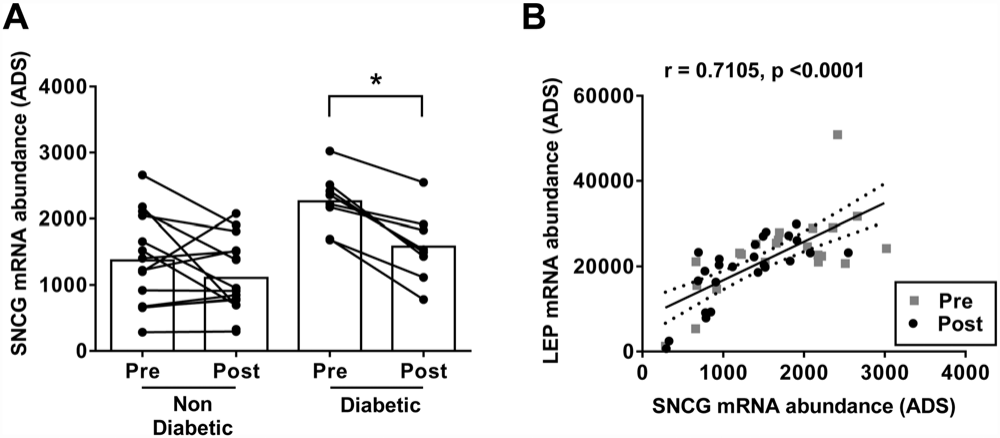
COHORT 1: 12 weeks PIO treatment significantly decreased synuclein-γ (SNCG) mRNA expression in subcutaneous white adipose tissue (SC-WAT) from pre-treatment levels in subjects with type 2 diabetes (n = 8) but not in non-diabetics (n = 17). **(A)**. *P<0.05, paired t test. Pre- and post-treatment SC-WAT expression levels of *SNCG* and *LEP* in the same subjects were highly-correlated in T2DM and non-diabetic subjects, using Pearson’s correlation statistic **(B)**. ADS—average difference score calculated using Affymetrix MAS5.0. Raw data is provided in S1 Data.

Using microarray results from a cohort of non-diabetic and type 2 diabetic PIO-treated individuals, treatment for 12 weeks significantly decreased subcutaneous WAT *SNCG* gene expression in type 2 diabetics by 30% (Fig. 3A). Non-diabetics did not show a significant decrease in WAT *SNCG* gene expression despite a downward trend in absolute terms (Fig. 3A). These observations suggest that PIO effects on subcutaneous WAT *SNCG* expression in humans manifests most clearly in the context of diabetes or with changes in glucose homeostasis. WAT *SNCG* and leptin (*LEP* gene) expression levels were strongly correlated at both the pre-treatment and post treatment time points for all subjects (Fig. 3B).

#### Cohort 2

**Fig 4:**
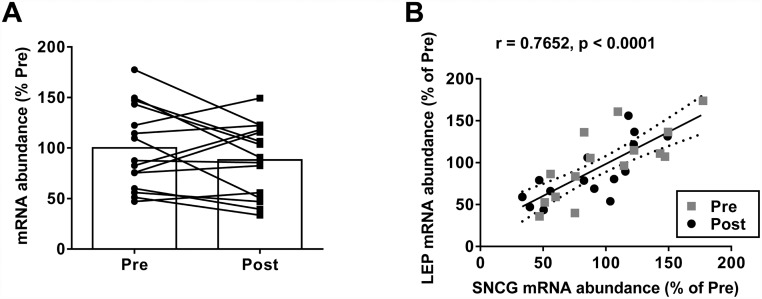
COHORT 2: Effect of 11 weeks PIO treatment on synuclein-γ (SNCG) mRNA expression in subcutaneous white adipose tissue (SC-WAT) in subjects with type 2 diabetes (n = 15). **(A)**. Pre- and post-treatment SC-WAT expression levels of *SNCG* and *LEP* in the same subjects were highly-correlated, using Pearson’s correlation statistic **(B)**. The average transcript level in pre-treatment biopsies was considered 100%. Raw data is provided in S1 Data.

In a second cohort of T2DM subjects, >11 weeks PIO treatment did not significantly decrease *SNCG* mRNA expression in subcutaneous WAT as measured by PCR analysis (100.0 ± 41.2% pre; 88.2 ± 35.8% post)(Fig. 4A). As previously reported for this cohort, other PPARγ target genes (*PEPCK1* and *LPL*) were only modestly impacted by PIO [21, 40]. *SNCG* and *LEP* WAT mRNA expression demonstrated a high degree of correlation both pre- and post-PIO treatment (Fig. 4B).

### Regulation of Sncg expression by rosiglitazone in murine primary dorsal root ganglia cell culture

**Fig 5:**
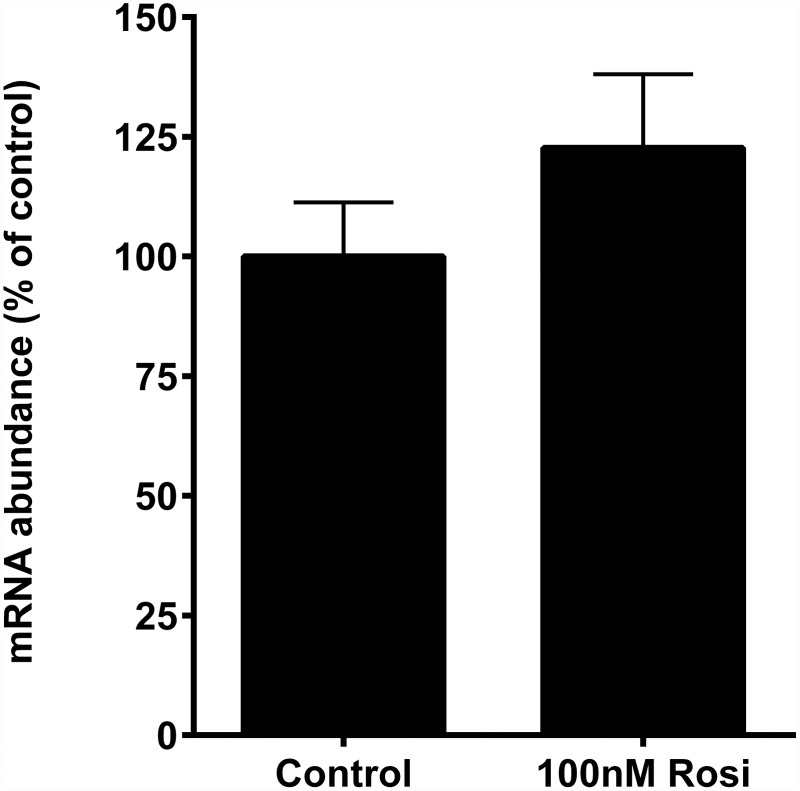
Effect of 24 hr treatment with 100 nM PPARγ agonist, rosiglitazone (Rosi), on mRNA expression levels for synuclein-γ (*Sncg*) in murine primary dorsal root ganglia neurons. Values are means +/- SEM, n = 17/treatment. Data are from four independent experiments. Transcript level in vehicle-treated control cells was considered 100%. Raw data is provided in S1 Data.

In addition to high expression in fat cells, synuclein-γ transcript abundance is robust in PNS neurons including those of the DRG [22, 49]. Considering the results in adipocytes and WAT supporting a regulatory role of PPARγ in modulating *Sncg* gene expression, we tested the hypothesis that TZD treatment of murine DRG neurons in culture would reduce *Sncg* transcript abundance. Freshly-isolated DRG neurons were exposed to vehicle or 100 nM rosiglitizone for 20 hr to mimic studies in adipocytes. To address well-to-well and preparation-to-preparation variation in cell type (DRG contain both neurons and accessory glial cells) which could confound interpretation of *Sncg* gene expression patterns, we measured mRNA abundance of calcitonin gene-related peptide (*Calca*), a classic marker for nociceptive DRG neurons. Rosiglitazone had no significant effect on *Calca* expression (100.0 ± 26.33% control; 120.4 ± 40.98% roziglitazone treated), and *Calca* expression was highly correlated with the 18S rRNA used as a total RNA loading control (r = 0.820; p<0.0001), indicating that the cell culture preparations were enriched with neurons. Rosiglitazone had no effect on *Sncg* mRNA expression (Fig. 5). When *Sncg* mRNA abundance was calculated using *Calca* as the control gene in lieu of 18S, rosiglitazone, likewise, showed no effect on *Sncg* expression. These results support the idea that the observations related to *Sncg* expression in the cell preparation reflected neurons in the culture.

## Discussion

Synuclein-γ is uniquely co-expressed in adipocytes and neurons yet its function and regulation are not fully elaborated, especially in terms of shared aspects between tissues. Recent findings implicate an important role for synuclein-γ in energy homeostasis and adipocyte function. For instance, whole-body *Sncg* knockout mice are resistant to diet-induced obesity (DIO) and exhibit increased adipocyte lipolysis and increased whole body lipid oxidation and energy expenditure [30]. This effect is attributed in part to synuclein-γ involvement in the formation of adipocyte SNARE complexes responsible for shuttling lipids into the adipocyte lipid droplet, but CNS or PNS effects could not be excluded especially in light of energy balance changes in the mice. In addition, synuclein-γ gene expression in WAT was found to be significantly increased in human obesity and up-regulated during the course of adipogenesis [22]. Synuclein-γ function in neurons is less understood, yet it is notable that it is structurally related to synuclein-α, which plays a role in neuronal synaptic vesicle fusion with cell membranes via SNARE complexes [50]. However, ablation of *Sncg* does not have any immediately obvious impact on neuronal development or function in mice [26]. *Sncg* total-body knockout mice do show alterations in lipid composition in the central nervous system (phosphatidylserine increased in the midbrain; increased levels of docosahexaenoic acid found in phosphatidylserine and phosphatidylethanolamine in the cerebral cortex) [31], suggesting involvement in neuronal lipid metabolism or trafficking. As more information is uncovered regarding synuclein-γ involvement in metabolism, it is important to understand the endocrinological or metabolic factors that regulate its transcription. The current studies add to the growing evidence that *Sncg* is regulated by the transcription factor PPARγ, a ligand-activated nuclear receptor important to adipocyte differentiation, energy storage, immune system regulation and metabolic homeostasis.

PPARγ forms a heterodimeric DNA-binding complex with RXRα, and activation by its endogenous ligands (including certain prostaglandins), or synthetic agonists (including the TZDs) lead to up- or down-regulation of gene expression [51]. A predicted PPARγ response element has been identified in the promoter region of the human *SNCG* gene [33], and previously we have demonstrated that *Sncg* transcript is decreased by the TZD PPARγ agonist troglitazone and the non-TZD GW1929 in cultured murine adipocytes [22]. Results of the current study now fully establish that the murine *Sncg* gene is a *bona fide* target of PPARγ in differentiated adipocytes. *Sncg* expression significantly decreased in mature 3T3-L1 adipocytes upon exposure to the TZD PPARγ agonist rosiglitazone, and co-treatment with rosiglitazone and a PPARγ antagonist (T0070907) diminished these effects. Rosiglitazone was used as a representative TZD that is responsive to T0070907 inhibition, and since we have shown that a variety of PPARγ agonists impact *Sncg* expression (i.e., rosiglitazone, troglitazone, GW1929), we do not believe that pioglitazone or other TZD or non-TZD agonists would behave differently on this outcome. Nevertheless, additional studies would be needed to validate this idea. Most importantly, ChIP data revealed PPARγ strongly binds to predicted PPAR-response elements of the murine *Sncg* gene in differentiating murine adipocytes.

We previously reported subcutaneous and visceral SC-WAT *SNCG* mRNA abundance to be highly correlated with *LEP* (leptin) mRNA in non-obese and obese women [22]. In the human cohorts examined herein, we again observed strong significant positive correlations, confirming shared regulatory elements for these two genes. Leptin expression and secretion corresponds to adiposity over a broad range in humans, and leptin is induced by glucose utilization [52], suggesting that higher leptin goes hand-in-hand with calorie storage and WAT growth. Considering the newly-discovered role for synuclein-γ in maintaining adipocyte lipid storage [30], it is therefore not surprising that WAT synuclein-γ expression is coordinately-regulated with that of leptin. TZDs promote the presence of smaller adipocytes and a multilocular triglyceride droplet phenotype [53, 54]. It is interesting to consider whether a reduction in synuclein-γ expression and activity under these conditions might play a role, considering the importance of synuclein-γ in lipid droplet formation and maintenance [30]. Such an idea will require further experimental testing to validate or refute.

Despite similar patterns of SNCG and LEP mRNA expression in subcutaneous WAT (SC-WAT) in humans, we discovered some divergence in the gene regulation of these factors in cultured murine 3T3-L1 adipocytes. PPARγ interacts with other nuclear receptors, including LXR, and PPARγ can both induce the expression of LXRα and increase activation of enzymes that produce endogenous LXR ligands [47]. Therefore, PPARγ activation can have both LXR-dependent and-independent actions. For example, PIO induces the mRNA expression of genes involved in macrophage cholesterol and phospholipid transport, ABCA1 and ABCG1, in either a LXR-dependent or-independent manner, respectively [55]. Studies herein using the LXR agonist, T0901317, indicated that TZD-associated reductions in *Sncg* expression were via LXR-independent mechanisms, unlike that of *Lep* that appeared to be mediated by LXR. The latter is consistent with prior limited observations for *Lep* (43). These diverging pathways for PPARγ action likely explained *Lep* and *Sncg* differential responses to the PPARγ antagonist, T0070907. At least two possibilities emerge. First, T0070907 modulates the ligand binding domain of PPARγ and therefore alters its interactions with cofactor proteins [36]. The nature of the PPARγ co-repressors and co-activators that regulate *Sncg* and *Fabp4* compared to *Lep* may differ, thus yielding differential responses to T0070907. Second, in our experimental conditions, adequate endogenous LXR agonists might have been present to maintain repression of *Lep* gene upon TZD treatment even in the presence of T0070907. This would argue that the antagonist does not impinge on TZD-activated generation of LXR agonist molecules in the adipocytes. These hypotheses warrant evaluation in future experiments.

We have demonstrated that *Sncg* expression can be regulated by TZDs through PPARγ activation in murine adipocytes in a cell-autonomous fashion, but until now it was unknown whether TZDs regulate WAT expression *in vivo*. In a cohort of non-diabetic and type 2 diabetic subjects representing a variety of BMIs, 12 week treatment with PIO modestly but significantly decreased subcutaneous WAT (SC-WAT) *SNCG* mRNA expression in type 2 diabetics but not in non-diabetics. Utilizing archived WAT samples from clinical studies of a different cohort of type 2 diabetics treated for >11 weeks with PIO, only a modest effect to decrease SC-WAT *SNCG* expression was observed. Possible reasons for the differences in the two cohorts/studies may be: differences in stage of disease progression across the cohorts, potential impact of non-TZD medication regimens, innate individual responsivity to TZD treatment, or sex-associated differences (Cohort 1 was predominantly male, and Cohort 2 had a majority of females). TZD-responsiveness is associated with transcriptional signatures in muscle and adipose, perhaps indicating a range in metabolic flexibility [39]. Taking these studies together, these results support the notion that transcriptional regulation of WAT SNCG *in vivo* by PPARγ is possible, but highlights the complicated nature of this relationship and high inter-individual variability. The possibility that TZDs impact subcutaneous WAT depots (used here) and visceral depots differently with respect to *SNCG* expression should also be considered, as should the potential confounder of differential adiposity gain with TZD treatment. The current studies were opportunistic in nature, which may have added to variability in SNCG responsiveness to PIO treatment. Future controlled clinical studies are warranted that are specifically designed to determine short- and long-term regulation of SNCG by PIO or other interventions that modify PPARγ activity in multiple WAT depots.

While the evidence indicates that synuclein-γ is a *bona fide* PPARγ target in adipocytes, nothing was previously known about the regulation of this gene in peripheral neurons, and little has been reported regarding PNS effects of PPARγ. The suggestion that PPARγ impacts PNS biology is supported by reports that TZD treatment improves gait and proprioception in T2D and insulin resistant subjects [14, 15], reduces neuropathic pain [56], and attenuates neuroinflammation in spinal cord injury models [19]. Abnormal accumulation of synuclein-γ protein in neurons and glial cells is associated with neurodengerative diseases such as Parkinson’s Disease [57], and TZDs have been demonstrated to have neuroprotective properties under such circumstances. In theory, these effects of TZDs could be associated with direct actions on PNS neurons to modulate target genes such as *Sncg*. However, our preliminary findings suggest that, unlike adipocytes, the *Sncg* gene is not regulated by PPARγ in adult mouse DRG, since the abundance of *Sncg* mRNA remained unaltered despite treatment of cultured neurons with rosiglitazone at a concentration maximally effective in cultured fat cells. One likely explanation for this finding is that *Sncg* is under tissue-specific regulation, consistent with different roles for the protein in various cell types. High levels of SNCG expression in breast cancer cells appears to be controlled by AP-1 regulatory sequences [58], however modifications by deletions or mutations in AP-1 binding sites of SNCG promoter affect its activity differentially in human neuroblastoma cells and human embryonic kidney cells [59]. With respect to synuclein-γ, since it is involved in lipid droplet formation and maintenance in adipocytes, perhaps it is not surprising that *Sncg* would be sensitive to PPARγ agonism in fat cells and not peripheral somatosensory neurons (not an important site for fuel storage).

Although PPARγ mRNA is detectable by PCR in DRG neurons ([18] and unpublished results), absolute levels are a tiny fraction of that in adipocytes (http://biogps.org; [60]). Very recently, Elmquist and colleagues reported that PPARγ levels are very low in DRG compared to vagal afferents [61], which might explain our lack of induction of *Sncg* mRNA in DRG neurons. An additional consideration is that regulation of the synuclein-γ gene by PPARγ may be isoform-specific: Alternative splicing and differential promoter usage results in two PPARγ isoforms, PPARγ1 (widely-expressed) and PPARγ2 (high in WAT and critical to adipocyte metabolic function) [62]. There is evidence that each isoform has unique gene regulatory functions in addition to overlapping actions [63, 64]. Should TZD-associated repression of *Sncg* expression require PPARγ2, it would explain the lack of effect of TZDs in PNS neurons. Supporting this view, we also observed that expression of Tusc5 mRNA (another adipocyte-PNS neuron-abundant PPARγ target gene product) was unaffected by TZD treatment in primary DRG neurons (data not shown). Robust induction of Tusc5 gene expression by TZD treatment was previously found to be PPARγ2-specific [21]. We acknowledge that a limitation of the current proof-of-principle study in DRG neurons is that only a single concentration of TZD was tested. While the concentration employed is maximally-effective on gene regulation in adipocytes, we cannot fully exclude the possibility that the EC_50_ in DRG neurons is much higher. Thus, additional work is needed to understand if PNS neuronal regulation of *Sncg* and other genes by TZDs and PPARγ is context-specific (e.g., related to neuronal age and plasticity state, concentration of ligand, type of PNS neuron) or dependent on PPARγ abundance, isoform type, or PPARγ cofactor patterns that differ from those in adipocytes.

In conclusion, our results prove that the *Sncg* gene is a PPARγ target in adipocytes, but there is a lack of evidence that this is also the case for DRG somatosensory neurons. In addition to the tissue-specific nature of this regulation, it is clear that inter-individual variability and metabolic status contribute to a complex *SNCG*-PPARγ relationship in humans. Also, despite strong correlations in human WAT synuclein-γ and leptin transcript expression *in vivo*, cell culture studies using a murine adipocyte model revealed nuanced differences in gene regulation, pointing to an LXR-independent nature of TZD-regulated *Sncg* expression in contrast to *Lep*. More work remains to map out the exact molecular events underlying *Sncg* gene regulation by PPARγ agonism in fat cells, and its similarities and differences from *Lep*. Altogether, our results showing correlative expression of synuclein-γ with leptin, and regulation of expression by the important metabolic gene regulator PPARγ, are consistent with the emerging view that synuclein-γ is a player in metabolic physiology and adipocyte fuel homeostasis.

## Supporting Information

S1 Data Raw data for Fig. 1, Fig. 3, Fig. 4, and Fig. 5. (XLS)

S1 Fig PCR promoter primers and respective amplicon sizes of-9.9 kb DR-1 site, −3.4 kb DR-1, and −6.2 kb DR-1. A representative image showing DNA band sizes is shown.
(TIF)

## References

[1] Song CK, Schwartz GJ, Bartness TJ (2009) Anterograde transneuronal viral tract tracing reveals central sensory circuits from white adipose tissue. Am J Physiol Regul Integr Comp Physiol 296: R501–511. 10.1152/ajpregu.90786.2008 19109367 PMC2665851

[2] Bartness TJ, Shrestha YB, Vaughan CH, Schwartz GJ, Song CK (2010) Sensory and sympathetic nervous system control of white adipose tissue lipolysis. Molecular and cellular endocrinology 318: 34–43. 10.1016/j.mce.2009.08.031 19747957 PMC2826518

[3] Bartness TJ, Vaughan CH, Song CK (2010) Sympathetic and sensory innervation of brown adipose tissue. Int J Obes (Lond) 34 Suppl 1: S36–42. 20935665 10.1038/ijo.2010.182 PMC3999344

[4] Vaughan CH, Shrestha YB, Bartness TJ (2011) Characterization of a novel melanocortin receptor-containing node in the SNS outflow circuitry to brown adipose tissue involved in thermogenesis. Brain research 1411: 17–27. 10.1016/j.brainres.2011.07.003 21802070 PMC3426614

[5] van Baak MA (2001) The peripheral sympathetic nervous system in human obesity. Obes Rev 2: 3–14. 12119635 10.1046/j.1467-789x.2001.00010.x

[6] Tentolouris N, Liatis S, Katsilambros N (2006) Sympathetic system activity in obesity and metabolic syndrome. Annals of the New York Academy of Sciences 1083: 129–152. 17148737 10.1196/annals.1367.010

[7] Raybould HE (2008) Nutrient sensing in the gastrointestinal tract: possible role for nutrient transporters. J Physiol Biochem 64: 349–356. 19391461 10.1007/BF03174091

[8] Paulino G, Barbier de la Serre C, Knotts TA, Oort PJ, Newman JW, et al (2009) Increased expression of receptors for orexigenic factors in nodose ganglion of diet-induced obese rats. Am J Physiol Endocrinol Metab 296: E898–903. 10.1152/ajpendo.90796.2008 19190260 PMC2670626

[9] de Lartigue G, Barbier de la Serre C, Espero E, Lee J, Raybould HE (2011) Diet-induced obesity leads to the development of leptin resistance in vagal afferent neurons. Am J Physiol Endocrinol Metab 301: E187–195. 10.1152/ajpendo.00056.2011 21521717 PMC3129833

[10] Davidson EP, Coppey LJ, Calcutt NA, Oltman CL, Yorek MA (2010) Diet-induced obesity in Sprague-Dawley rats causes microvascular and neural dysfunction. Diabetes Metab Res Rev 26: 306–318. 10.1002/dmrr.1088 20503263 PMC2878284

[11] Obrosova IG, Ilnytska O, Lyzogubov VV, Pavlov IA, Mashtalir N, et al (2007) High-fat diet induced neuropathy of pre-diabetes and obesity: effects of “healthy” diet and aldose reductase inhibition. Diabetes 56: 2598–2608. 17626889 10.2337/db06-1176

[12] Dunn TN, Adams SH (2014) Relations between Metabolic Homeostasis, Diet, and Peripheral Afferent Neuron Biology. Advances in nutrition 5: 386–393. 10.3945/an.113.005439 25022988 PMC4085187

[13] Tontonoz P, Spiegelman BM (2008) Fat and beyond: the diverse biology of PPARgamma. Annu Rev Biochem 77: 289–312. 10.1146/annurev.biochem.77.061307.091829 18518822

[14] Petrofsky J, Lee S, Cuneo ML (2005) Gait characteristics in patients with type 2 diabetes; improvement after administration of rosiglitazone. Med Sci Monit 11: PI43–51. 15917728

[15] Petrofsky JS, Lee S, Cuneo-Libarona M (2005) The impact of rosiglitazone on heat tolerance in patients with type 2 diabetes. Med Sci Monit 11: CR562–569. 16319786

[16] Carta AR, Frau L, Pisanu A, Wardas J, Spiga S, et al (2011) Rosiglitazone decreases peroxisome proliferator receptor-gamma levels in microglia and inhibits TNF-alpha production: new evidences on neuroprotection in a progressive Parkinson’s disease model. Neuroscience 194: 250–261. 10.1016/j.neuroscience.2011.07.046 21839812

[17] Kapadia R, Yi JH, Vemuganti R (2008) Mechanisms of anti-inflammatory and neuroprotective actions of PPAR-gamma agonists. Frontiers in bioscience: a journal and virtual library 13: 1813–1826. 17981670 10.2741/2802 PMC2734868

[18] Maeda T, Kiguchi N, Kobayashi Y, Ozaki M, Kishioka S (2008) Pioglitazone attenuates tactile allodynia and thermal hyperalgesia in mice subjected to peripheral nerve injury. Journal of pharmacological sciences 108: 341–347. 19008646 10.1254/jphs.08207fp

[19] Park SW, Yi JH, Miranpuri G, Satriotomo I, Bowen K, et al (2007) Thiazolidinedione class of peroxisome proliferator-activated receptor gamma agonists prevents neuronal damage, motor dysfunction, myelin loss, neuropathic pain, and inflammation after spinal cord injury in adult rats. The Journal of pharmacology and experimental therapeutics 320: 1002–1012. 17167171 10.1124/jpet.106.113472

[20] Lu M, Sarruf DA, Talukdar S, Sharma S, Li P, et al (2011) Brain PPAR-gamma promotes obesity and is required for the insulin-sensitizing effect of thiazolidinediones. Nat Med 17: 618–622. 10.1038/nm.2332 21532596 PMC3380629

[21] Knotts TA, Lee HW, Kim JB, Oort PJ, McPherson R, et al (2009) Molecular Characterization of the Tumor Suppressor Candidate 5 Gene: Regulation by PPARgamma and Identification of TUSC5 Coding Variants in Lean and Obese Humans. PPAR Res 2009: 867678 10.1155/2009/867678 20204174 PMC2830574

[22] Oort PJ, Knotts TA, Grino M, Naour N, Bastard JP, et al (2008) Gamma-synuclein is an adipocyte-neuron gene coordinately expressed with leptin and increased in human obesity. J Nutr 138: 841–848. 18424589 10.1093/jn/138.5.841 PMC3160639

[23] Oort PJ, Warden CH, Baumann TK, Knotts TA, Adams SH (2007) Characterization of Tusc5, an adipocyte gene co-expressed in peripheral neurons. Molecular and cellular endocrinology 276: 24–35. 17689857 10.1016/j.mce.2007.06.005

[24] Buchman VL, Adu J, Pinon LG, Ninkina NN, Davies AM (1998) Persyn, a member of the synuclein family, influences neurofilament network integrity. Nat Neurosci 1: 101–103. 10195122 10.1038/349

[25] Lavedan C, Leroy E, Torres R, Dehejia A, Dutra A, et al (1998) Genomic organization and expression of the human beta-synuclein gene (SNCB). Genomics 54: 173–175. 9806846 10.1006/geno.1998.5556

[26] Ninkina N, Papachroni K, Robertson DC, Schmidt O, Delaney L, et al (2003) Neurons expressing the highest levels of gamma-synuclein are unaffected by targeted inactivation of the gene. Molecular and cellular biology 23: 8233–8245. 14585981 10.1128/MCB.23.22.8233-8245.2003 PMC262405

[27] Jia T, Liu YE, Liu J, Shi YE (1999) Stimulation of breast cancer invasion and metastasis by synuclein gamma. Cancer research 59: 742–747. 9973226

[28] Surgucheva IG, Sivak JM, Fini ME, Palazzo RE, Surguchov AP (2003) Effect of gamma-synuclein overexpression on matrix metalloproteinases in retinoblastoma Y79 cells. Archives of biochemistry and biophysics 410: 167–176. 12559990 10.1016/s0003-9861(02)00664-1

[29] Willis D, Li KW, Zheng JQ, Chang JH, Smit AB, et al (2005) Differential transport and local translation of cytoskeletal, injury-response, and neurodegeneration protein mRNAs in axons. The Journal of neuroscience: the official journal of the Society for Neuroscience 25: 778–791. 15673657 10.1523/JNEUROSCI.4235-04.2005 PMC6725618

[30] Millership S, Ninkina N, Guschina IA, Norton J, Brambilla R, et al (2012) Increased lipolysis and altered lipid homeostasis protect gamma-synuclein-null mutant mice from diet-induced obesity. Proceedings of the National Academy of Sciences of the United States of America 109: 20943–20948. 10.1073/pnas.1210022110 23213245 PMC3529034

[31] Guschina I, Millership S, O’Donnell V, Ninkina N, Harwood J, et al (2011) Lipid classes and fatty acid patterns are altered in the brain of gamma-synuclein null mutant mice. Lipids 46: 121–130. 10.1007/s11745-010-3486-0 20963507 PMC3038238

[32] Tung YC, Ma M, Piper S, Coll A, O’Rahilly S, et al (2008) Novel leptin-regulated genes revealed by transcriptional profiling of the hypothalamic paraventricular nucleus. The Journal of neuroscience: the official journal of the Society for Neuroscience 28: 12419–12426. 10.1523/JNEUROSCI.3412-08.2008 19020034 PMC2650686

[33] Lemay DG, Hwang DH (2006) Genome-wide identification of peroxisome proliferator response elements using integrated computational genomics. J Lipid Res 47: 1583–1587. 16585784 10.1194/jlr.M500504-JLR200

[34] Ross SE, Erickson RL, Gerin I, DeRose PM, Bajnok L, et al (2002) Microarray analyses during adipogenesis: understanding the effects of Wnt signaling on adipogenesis and the roles of liver X receptor alpha in adipocyte metabolism. Molecular and cellular biology 22: 5989–5999. 12138207 10.1128/MCB.22.16.5989-5999.2002 PMC133961

[35] Juvet LK, Andresen SM, Schuster GU, Dalen KT, Tobin KA, et al (2003) On the role of liver X receptors in lipid accumulation in adipocytes. Mol Endocrinol 17: 172–182. 12554745 10.1210/me.2001-0210

[36] Lee G, Elwood F, McNally J, Weiszmann J, Lindstrom M, et al (2002) T0070907, a selective ligand for peroxisome proliferator-activated receptor gamma, functions as an antagonist of biochemical and cellular activities. The Journal of biological chemistry 277: 19649–19657. 11877444 10.1074/jbc.M200743200

[37] Seo JB, Noh MJ, Yoo EJ, Park SY, Park J, et al (2003) Functional characterization of the human resistin promoter with adipocyte determination- and differentiation-dependent factor 1/sterol regulatory element binding protein 1c and CCAAT enhancer binding protein-alpha. Mol Endocrinol 17: 1522–1533. 12730330 10.1210/me.2003-0028

[38] Lee YS, Sohn DH, Han D, Lee HW, Seong RH, et al (2007) Chromatin remodeling complex interacts with ADD1/SREBP1c to mediate insulin-dependent regulation of gene expression. Molecular and cellular biology 27: 438–452. 17074803 10.1128/MCB.00490-06 PMC1800793

[39] Sears DD, Hsiao G, Hsiao A, Yu JG, Courtney CH, et al (2009) Mechanisms of human insulin resistance and thiazolidinedione-mediated insulin sensitization. Proceedings of the National Academy of Sciences of the United States of America 106: 18745–18750. 10.1073/pnas.0903032106 19841271 PMC2763882

[40] Bogacka I, Xie H, Bray GA, Smith SR (2004) The effect of pioglitazone on peroxisome proliferator-activated receptor-gamma target genes related to lipid storage in vivo. Diabetes care 27: 1660–1667. 15220243 10.2337/diacare.27.7.1660

[41] Akiyama T, Tominaga M, Davoodi A, Nagamine M, Blansit K, et al (2012) Cross-sensitization of histamine-independent itch in mouse primary sensory neurons. Neuroscience 226: 305–312. 10.1016/j.neuroscience.2012.09.019 23000623 PMC3489980

[42] Fuenzalida K, Quintanilla R, Ramos P, Piderit D, Fuentealba RA, et al (2007) Peroxisome proliferator-activated receptor gamma up-regulates the Bcl-2 anti-apoptotic protein in neurons and induces mitochondrial stabilization and protection against oxidative stress and apoptosis. The Journal of biological chemistry 282: 37006–37015. 17965419 10.1074/jbc.M700447200

[43] Tontonoz P, Hu E, Spiegelman BM (1994) Stimulation of adipogenesis in fibroblasts by PPAR gamma 2, a lipid-activated transcription factor. Cell 79: 1147–1156. 8001151 10.1016/0092-8674(94)90006-x

[44] Hu E, Liang P, Spiegelman BM (1996) AdipoQ is a novel adipose-specific gene dysregulated in obesity. The Journal of biological chemistry 271: 10697–10703. 8631877 10.1074/jbc.271.18.10697

[45] Hammarstedt A, Andersson CX, Rotter Sopasakis V and Smith U (2005) The effect of PPARgamma ligands on the adipose tissue in insulin resistance. Prostaglandins, leukotrienes, and essential fatty acids 73: 65–75. 15936183 10.1016/j.plefa.2005.04.008

[46] Giguere V (1999) Orphan nuclear receptors: from gene to function. Endocr Rev 20: 689–725. 10529899 10.1210/edrv.20.5.0378

[47] Steffensen KR, Gustafsson JA (2004) Putative metabolic effects of the liver X receptor (LXR). Diabetes 53 Suppl 1: S36–42. 14749264 10.2337/diabetes.53.2007.s36

[48] Chawla A, Boisvert WA, Lee CH, Laffitte BA, Barak Y, et al (2001) A PPAR gamma-LXR-ABCA1 pathway in macrophages is involved in cholesterol efflux and atherogenesis. Mol Cell 7: 161–171. 11172721 10.1016/s1097-2765(01)00164-2

[49] Buchman VL, Hunter HJ, Pinon LG, Thompson J, Privalova EM, et al (1998) Persyn, a member of the synuclein family, has a distinct pattern of expression in the developing nervous system. The Journal of neuroscience: the official journal of the Society for Neuroscience 18: 9335–9341. 9801372 10.1523/JNEUROSCI.18-22-09335.1998 PMC6792889

[50] Burre J, Sharma M, Tsetsenis T, Buchman V, Etherton MR, et al (2010) Alpha-synuclein promotes SNARE-complex assembly in vivo and in vitro. Science 329: 1663–1667. 10.1126/science.1195227 20798282 PMC3235365

[51] Nagy L, Szanto A, Szatmari I, Szeles L (2012) Nuclear hormone receptors enable macrophages and dendritic cells to sense their lipid environment and shape their immune response. Physiol Rev 92: 739–789. 10.1152/physrev.00004.2011 22535896

[52] Wellhoener P, Fruehwald-Schultes B, Kern W, Dantz D, Kerner W, et al (2000) Glucose metabolism rather than insulin is a main determinant of leptin secretion in humans. The Journal of clinical endocrinology and metabolism 85: 1267–1271. 10720074 10.1210/jcem.85.3.6483

[53] de Souza CJ, Eckhardt M, Gagen K, Dong M, Chen W, et al (2001) Effects of pioglitazone on adipose tissue remodeling within the setting of obesity and insulin resistance. Diabetes 50: 1863–1871. 11473050 10.2337/diabetes.50.8.1863

[54] Petrovic N, Walden TB, Shabalina IG, Timmons JA, Cannon B, et al (2009) Chronic peroxisome proliferator-activated receptor gamma (PPARgamma) activation of epididymally derived white adipocyte cultures reveals a population of thermogenically competent, UCP1-containing adipocytes molecularly distinct from classic brown adipocytes. The Journal of biological chemistry 285: 7153–7164. 10.1074/jbc.M109.053942 20028987 PMC2844165

[55] Ozasa H, Ayaori M, Iizuka M, Terao Y, Uto-Kondo H, et al (2011) Pioglitazone enhances cholesterol efflux from macrophages by increasing ABCA1/ABCG1 expressions via PPARgamma/LXRalpha pathway: findings from in vitro and ex vivo studies. Atherosclerosis 219: 141–150. 10.1016/j.atherosclerosis.2011.07.113 21862012

[56] Iwai S, Maeda T, Kiguchi N, Kobayashi Y, Fukazawa Y, et al (2008) Pioglitazone attenuates tactile allodynia and microglial activation in mice with peripheral nerve injury. Drug discoveries & therapeutics 2: 353–356. 22504745

[57] Galvin JE, Uryu K, Lee VM, Trojanowski JQ (1999) Axon pathology in Parkinson’s disease and Lewy body dementia hippocampus contains alpha-, beta-, and gamma-synuclein. Proceedings of the National Academy of Sciences of the United States of America 96: 13450–13455. 10557341 10.1073/pnas.96.23.13450 PMC23968

[58] Lu A, Zhang F, Gupta A, Liu J (2002) Blockade of AP1 transactivation abrogates the abnormal expression of breast cancer-specific gene 1 in breast cancer cells. The Journal of biological chemistry 277: 31364–31372. 12072430 10.1074/jbc.M201060200

[59] Surgucheva I, Surguchov A (2008) Gamma-synuclein: cell-type-specific promoter activity and binding to transcription factors. J Mol Neurosci 35: 267–271. 10.1007/s12031-008-9074-6 18498014

[60] Wu C, Orozco C, Boyer J, Leglise M, Goodale J, et al (2009) BioGPS: an extensible and customizable portal for querying and organizing gene annotation resources. Genome Biol 10: R130 10.1186/gb-2009-10-11-r130 19919682 PMC3091323

[61] Liu C, Bookout AL, Lee S, Sun K, Jia L, et al (2014) PPAR gamma in Vagal Neurons Regulates High-Fat Diet Induced Thermogenesis. Cell Metab 19: 722–730. 10.1016/j.cmet.2014.01.021 24703703 PMC4046333

[62] van Beekum O, Fleskens V, Kalkhoven E (2009) Posttranslational modifications of PPAR-gamma: fine-tuning the metabolic master regulator. Obesity (Silver Spring) 17: 213–219. 10.1038/oby.2008.473 19169221

[63] Werman A, Hollenberg A, Solanes G, Bjorbaek C, Vidal-Puig AJ, et al (1997) Ligand-independent activation domain in the N terminus of peroxisome proliferator-activated receptor gamma (PPARgamma). Differential activity of PPARgamma1 and-2 isoforms and influence of insulin. The Journal of biological chemistry 272: 20230–20235. 9242701 10.1074/jbc.272.32.20230

[64] Strand DW, Jiang M, Murphy TA, Yi Y, Konvinse KC, et al (2012) PPARgamma isoforms differentially regulate metabolic networks to mediate mouse prostatic epithelial differentiation. Cell Death Dis 3: e361 10.1038/cddis.2012.99 22874998 PMC3434663

