# Supplementary figures and images for: Evaluation of the Synuclein-γ (SNCG) Gene as a PPARγ Target in Murine Adipocytes, Dorsal Root Ganglia Somatosensory Neurons, and Human Adipose Tissue
Source: PLoS One. 2015 Mar 10;10(3):e0115830. doi: 10.1371/journal.pone.0115830 (PMC4355072; doi:10.1371/journal.pone.0115830)

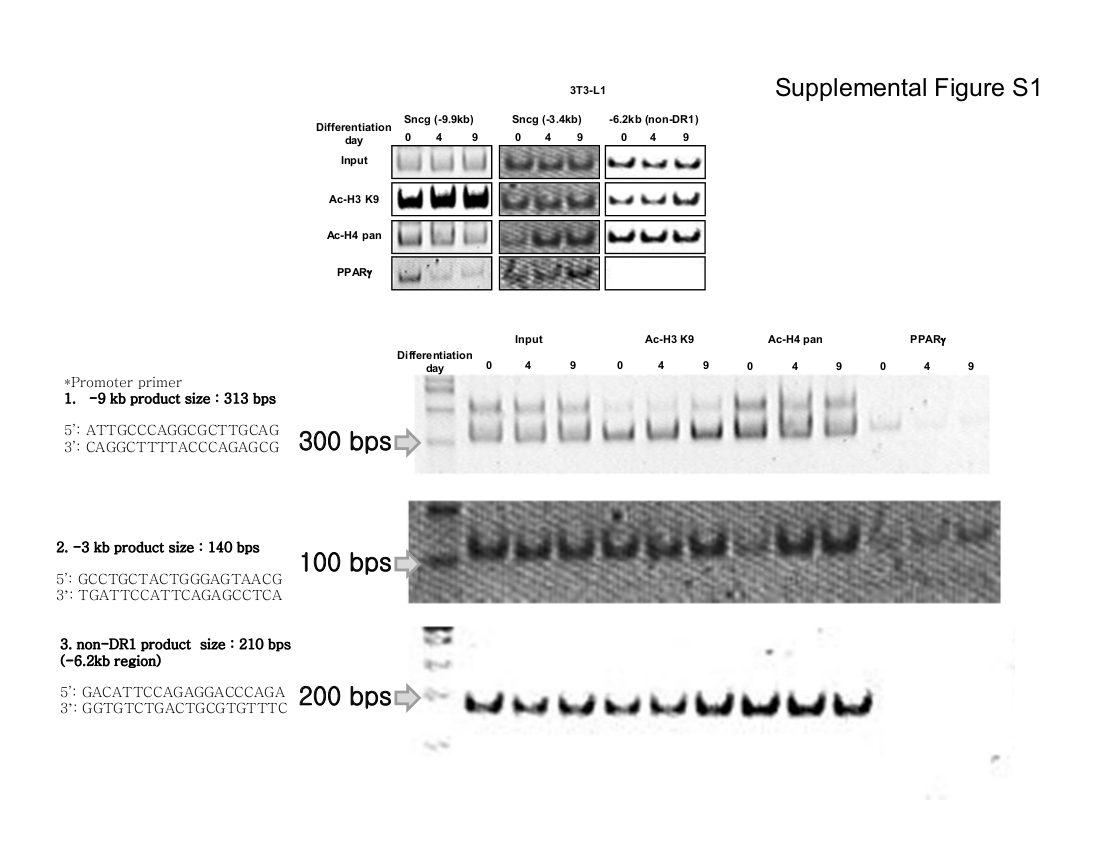

Supplement: S1 Fig — A representative image showing DNA band sizes is shown. (TIF) [file pone.0115830.s002.tif]
